# Inflammation and Oxidative Stress Induced by Obesity, Gestational Diabetes, and Preeclampsia in Pregnancy: Role of High-Density Lipoproteins as Vectors for Bioactive Compounds

**DOI:** 10.3390/antiox12101894

**Published:** 2023-10-23

**Authors:** Angélica Saraí Jiménez-Osorio, Elizabeth Carreón-Torres, Emmanuel Correa-Solís, Julieta Ángel-García, José Arias-Rico, Octavio Jiménez-Garza, Lizbeth Morales-Castillejos, Hugo Alexander Díaz-Zuleta, Rosa María Baltazar-Tellez, María Luisa Sánchez-Padilla, Olga Rocío Flores-Chávez, Diego Estrada-Luna

**Affiliations:** 1 Área Académica de Enfermería, Instituto de Ciencias de la Salud, Universidad Autónoma del Estado Hida go, Circuito Ex Hacienda La Concepción S/N, Carretera Pachuca-Actopan, San Agustín Tlaxiaca 42160, Hidalgo, Mexico; angelica_jimenez@uaeh.edu.mx (A.S.J.-O.); julieta_angel@uaeh.edu.mx (J.Á.-G.); jose_arias@uaeh.edu.mx (J.A.-R.); octavio_jimenez@uaeh.edu.mx (O.J.-G.); lizbeth_morales@uaeh.edu.mx (L.M.-C.); rosa_baltazar@uaeh.edu.mx (R.M.B.-T.); maria_sanchez2789@uaeh.edu.mx (M.L.S.-P.); ofloresc@uaeh.edu.mx (O.R.F.-C.); 2 Department of Molecular Biology, Instituto Nacional de Cardiología “Ignacio Chávez”, Juan Badiano 1, Sección XVI, Tlalpan, Mexico City 14080, Mexico; qfbelizabethcm@yahoo.es; 3 Instituto de Farmacobiología, Universidad de la Cañada, Carretera Teotitlán-San Antonio Nanahuatipán Km 1.7 s/n., Paraje Titlacuatitla, Teotitlán de Flores Magón 68540, Oaxaca, Mexico; emmanuelcs@unca.edu.mx; 4 Facultad de Ciencias de la Salud, Universidad de Ciencias Aplicadas y Ambientales, Cl. 222 #54-21, Bogotá 111166, Colombia; hdiazz@udca.edu.co

**Keywords:** inflammation, oxidative stress, pregnancy, high-density lipoproteins, bioactive compounds

## Abstract

Inflammation and oxidative stress are essential components in a myriad of pathogenic entities that lead to metabolic and chronic diseases. Moreover, inflammation in its different phases is necessary for the initiation and maintenance of a healthy pregnancy. Therefore, an equilibrium between a necessary/pathologic level of inflammation and oxidative stress during pregnancy is needed to avoid disease development. High-density lipoproteins (HDL) are important for a healthy pregnancy and a good neonatal outcome. Their role in fetal development during challenging situations is vital for maintaining the equilibrium. However, in certain conditions, such as obesity, diabetes, and other cardiovascular diseases, it has been observed that HDL loses its protective properties, becoming dysfunctional. Bioactive compounds have been widely studied as mediators of inflammation and oxidative stress in different diseases, but their mechanisms of action are still unknown. Nonetheless, these agents, which are obtained from functional foods, increase the concentration of HDL, TRC, and antioxidant activity. Therefore, this review first summarizes several mechanisms of HDL participation in the equilibrium between inflammation and oxidative stress. Second, it gives an insight into how HDL may act as a vector for bioactive compounds. Third, it describes the relationships between the inflammation process in pregnancy and HDL activity. Consequently, different databases were used, including MEDLINE, PubMed, and Scopus, where scientific articles published in the English language up to 2023 were identified.

## 1. Introduction

Pregnancy is a physiological process that extends the development phase of the fetus in the uterus. In this period, women experience a series of adjustments to satisfy the metabolic, biochemical, endocrine, and cellular changes that fetuses demand [1]. Nevertheless, depending on the woman’s wellness, these adaptations may be tolerated or be a burden in the development of certain diseases [2], such as obesity, hypertension, hyperlipidemia, metabolic syndrome, diabetes mellitus, preeclampsia, and preterm birth, which in turn, increases the risk of cardiovascular diseases and may even produce death [3,4]. Also, these metabolic disorders are characterized by the presence of endothelial dysfunction, inflammatory, pro-oxidant, and lipid profile dysregulation processes that increase the levels of total cholesterol, triglycerides, low-density lipoprotein cholesterol (c-LDL), and dysfunctional high-density lipoproteins (HDL) [5,6]. Obesity, diabetes, and preeclampsia induce oxidative stress conditions on their own through a number of mechanisms or pathways, including superoxide anion generation, protein kinase-C activation, hyperglycemia, elevated cholesterol and triglyceride levels, mitochondrial dysfunction, low antioxidant system, chronic inflammation, and reactive oxygen species (ROS) generation, especially during the postprandial period [7,8,9,10]. In pregnancy, these mechanisms exacerbate oxidative stress (OS) and inflammation processes with deleterious effects on the fetus and child’s development, along with the damage to the mother.

Foods, fruits, herbs, and seeds are widely used in diverse cultures for the acute treatment of symptoms related to pain or inflammation, and in a long-term fashion to control the negative effects of some chronic diseases. Their method of consumption passes from one generation to the next, and it is part of several cultures and customs of diverse societies [11,12,13]. In some cultures, the consumption of plants during pregnancy is common. For example, plants are used to treat nausea, vomiting, or dizziness; nonetheless, their effectiveness in chronic degenerative diseases experienced by pregnant women has not been fully elucidated [14,15]. Therefore, the purpose of this review is to provide the most recent and relevant information concerning the role high-density lipoproteins play as vectors of bioactive compounds with a protective role in the processes of inflammation and OS involved in metabolic diseases that are frequently present during pregnancy, such as gestational diabetes, obesity, and preeclampsia.

## 2. Methods

This narrative review was carried out by considering scientific articles of interest for the topic, apprising those related to pregnancy and inflammation, and pregnancy and oxidative stress. These processes were considered factors for predisposition to chronic diseases for the mother and the fetus during the gestational stages such as childhood and adulthood. As selection criteria, the following descriptors were used: inflammation, oxidative stress, pregnancy, high-density lipoproteins, metabolic diseases, and bioactive compounds. To search for primary and secondary information sources, electronic databases such as MEDLINE, PubMed, and Scopus were used, finding original papers published from 1982 to August 2023. Selected articles for bibliographic research were categorized by clinical relevance, short-term (prematurity, preeclampsia, gestational diabetes) and long-term (diabetes mellitus, obesity, dyslipidemia) outcome term, type of population (animal models, humans) that was used, outcomes of the behavior of the inflammatory markers that were stated, and the effect of bioactive compounds on lipoproteins during pregnancy. Ultimately, a total of 207 references were selected, which were available in full text and in the English language.

## 3. Bioactive Compounds Contained in Foods Used during Pregnancy

Over the course of human history, some herbs, plants, and fruits have been used extensively in different cultures and societies to treat injuries, diseases, viruses, and bacterial infections, and even to enhance women’s health during pregnancy to facilitate childbirth [16]. In pregnancy, the particular use of plants and herbs is focused mostly on alleviating conditions, such as diarrhea, chills, nausea, constipation, vomiting, or to increase milk production and accelerate labor. Several studies have reported that the mentioned capabilities are attributed to the bioactive compounds contained in herbs, fruits, spices, and other foods possessing anti-inflammatory [17], antimicrobial [18], antioxidant [19], cardioprotective [20], hepatoprotective [21], hypoglycemic [22], neuroprotective [23], immunomodulatory [24], or hypolipidemic [25] properties. Some of these bioactive compounds include phytochemicals, phenolic compounds, polysaccharides, and unsaturated fatty acids. During pregnancy, the consumption and functionality of these compounds have been established [26], especially in the prevention of diseases and health promotion for both mother and fetus in the different phases of the gestational period [27,28,29].

The consumption of certain polyphenolic compounds in pregnancy improved levels of blood glucose in women with gestational diabetes [30], as well as insulin secretion in the fetus and in the mother [31]. Furthermore, it has been reported that some polyphenolic compounds regulate cell apoptosis, the secretion of adipokines, placentation, and arterial pressure. Such regulation occurs mainly through the activity of the matrix metalloproteinases (MMPs) [32,33] and some glucose transporter (GLUT) family members (GLUT1, GLUT4, and GLUT 9) [34].

A wide variety of secondary metabolites including flavonoids, quinones, flavones, tannins, and aromatic compounds are consumed in high frequency and quantity. Nonetheless, they could present a toxic effect mainly on the fetus because some of those metabolites are widely consumed during the first trimester. In fact, some of them are well known to be potentially dangerous because of the vulnerability of the fetus, for example, Japanese mint (*Mentha arvensis*), linden herb (*Justicia pectoralis*), verbena (*Verbena officinalis*), aloe (*Aloe vera*), chamomile (*Matricaria recutita*), oregano (*Plecthranthus amboinicus*), basil (*Ocicum basilicum*), caña santa (*Cymbopoggon citratus*), and wormwood (*Artemisia abssinthium*). Furthermore, some of these compounds could increase estrogenic activity by stimulating uterine contractility, increasing blood pressure, and having a hepatotoxic, mutagenic, or fetal malformation effect. Most of these agents may also have an effect on OS and inflammatory processes present in gestational diabetes mellitus (GDM), preeclampsia, or obesity during pregnancy because they contain phenolic compounds, flavonoids, tannins, omega-3, quercetin, kaempferol, tocopherols, and vitamin C [35,36,37,38,39,40].

## 4. Pregnancy–Oxidative Stress–Inflammation Association

A significant increase in inflammatory processes and OS markers during pregnancy happens because of biological actions that are carried out to maintain body homeostasis. The first reports showed an elevation in OS and inflammatory markers in plasma and urine during the physiological course of pregnancy [41,42]. The significant increase in inflammatory and pro-oxidant molecules can trigger negative conditions such as preeclampsia, endothelial dysfunction, pre-diabetes, poor fetal development, or premature birth [43,44].

One of the reasons for the increase in OS is the development of the placenta, which contains high quantities of mitochondria, leading to an increase in the concentration of ROS, such as superoxide dismutase (SOD), xanthine oxidase (XO), or NADPH oxidase (NOX). The high activity of these prooxidant enzymes is associated with the onset of preeclampsia, which is one of the most common negative conditions during pregnancy, as well as an increase in cholesterol, triglycerides, and c-LDL levels [45,46]. Nevertheless, a number of external factors are related to OS augmentation during pregnancy, such as smoking, consumption of foods rich in saturated fatty acids, carbohydrates, alcohol consumption, ultraviolet radiation, obesity, and consumption of non-steroidal anti-inflammatory drugs like paracetamol, ibuprofen, or diclofenac [47,48].

ROS play a crucial role throughout the entire process of pregnancy. It starts with the generation of steroid hormones in the ovary, oocyte maturation, ovulation, luteolysis, implantation, follicular growth, and maintenance of pregnancy until childbirth [49]. Elevated ROS production leads to a decreased ovarian blood flow and tissue damage, polycystic ovarian syndrome, or endometriosis. In addition, inflammatory processes are important and play an important role in both the positive and negative aspects, alongside important functions of the adaptive immune system; during the first and third trimester, there is a presence of pro-inflammatory processes, triggered by implantation and placentation, as well as labor and delivery, whereas, in the second trimester, anti-inflammatory processes proliferate [50,51], ensuring proper fetal growth at this stage.

**Figure 1:**
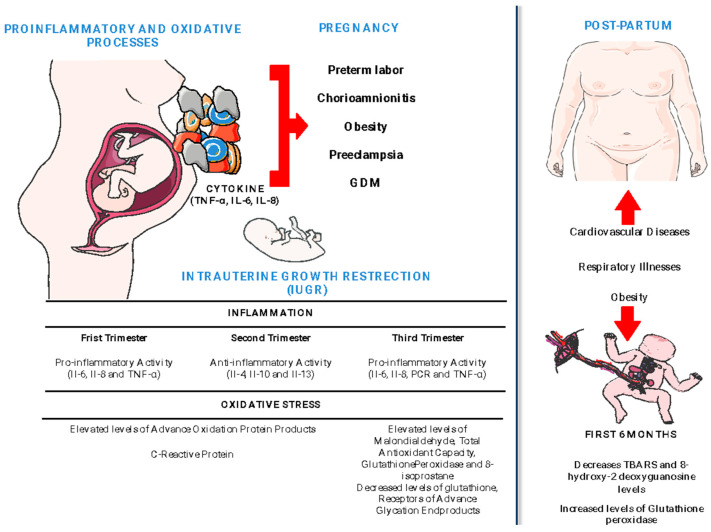
Inflammation and OS processes during and after pregnancy.

During gestation, women’s bodies possess diverse hormones, cytokines, nutrients, and enzymes that help maintain the balance of antioxidant and inflammatory processes (Figure 1) [52]. Advanced oxidation protein products (AOPPs) and C-reactive protein (CRP) levels in the maternal serum of pregnant women were significantly higher in the first and second trimesters compared to non-pregnant women. However, higher levels of AOPP were found in control men than in control non-pregnant women and were similar to pregnant women. Moreover, the CRP concentrations increased gradually from the first to the second trimester [38]. In contrast, an increase in OS was observed in the second trimester. The activity of the antioxidant enzyme glutathione peroxidase (GPx) was lower in pregnant women than non-pregnant women and postpartum women. Also, the oxidative damage marker to DNA, 8-hydroxy-2′-deoxyguanosine (8-OHdG), was higher in pregnant women compared to non-pregnant women [53].

During the third gestational stage, healthy pregnant women showed higher lipid peroxidation markers, such as malondialdehyde (MDA) in plasma and erythrocytes, than non-pregnant women, as well as lower levels of erythrocyte glutathione (GSH) levels than non-pregnant women [41]. Furthermore, a longitudinal study consisting of pregnant women at 30 weeks of gestational age showed that, after delivery, their products decreased levels of Ne-carboxymethyl lysine. Also, a soluble receptor for advanced glycation end products (sRAGE) was observed without changes in tumor necrosis factor alpha (TNF-α), CRP, and MDA in blood samples. Their newborns were healthy, but the newborns’ levels of sRAGE, TNF-α, and MDA were higher than their mothers at delivery [54]. Additionally, factors such as age and race influence OS and inflammation. Older pregnant women (age > 40 years) with uncomplicated pregnancies had lower anti-inflammatory markers, such as interleukin-10 (IL-10) and interleukin-1 receptor antagonist (IL-RA), and an increase in total antioxidant capacity (TAC) than younger pregnant women [55]. In Nigerian pregnant women, levels of vitamin E, an endogenous antioxidant, were observed to be lower than in pregnant women from the USA. However, levels of vitamin E depend on the isoform. Thus, in the US population, higher maternal α-tocopherol levels were associated with birth length (>10th percentile), while higher cord levels of α-tocopherol were associated with birth length (<10th percentile); these results were not observed in the Nigerian population, although the levels of delta-tocopherol were higher than maternal samples from the USA [56].

Undoubtedly, OS and inflammation are major factors in the development of diseases that are considered the first cause of death and morbidity in women during pregnancy since they can exacerbate negative conditions for the development of cardiovascular diseases and diabetes [3,57,58].

The inflammatory response during pregnancy is caused by toll-like receptor (TLR) activation, while inducing the activation of the inflammasome complex, to perpetuate the production of cytokines and chemokines in placenta and endometrium, such as monocyte chemotactic protein 1 (MCP-1), IL-8, IL1-β, and IL-6. This TLR activation triggers immune cell recruitment and the production of prostaglandins and MMPs, which leads to cervical cell activation and uterine contractions [59]. According to the stage of pregnancy, in vaginal epithelium, exocervix, endocervix, endometrium, and fallopian tubes, TLRs 1–5 induce the release of cytokines, chemokines, and antimicrobial peptides that modulate both proinflammatory and anti-inflammatory responses. Hence, it can promote or attenuate the onset of chronic diseases or adverse clinical conditions (Table 1) [60].

The mechanisms of inflammation suggested at this stage of life involve the role of type 1 and 2 T Helper (Th) cells, where Th1 cells are responsible for increased levels of interferon gamma (IFN-γ), interleukin 2 (IL-2), and tumor necrosis factor beta (TNF-β), which are responsible for phagocyte-dependent inflammation, as well as protection against intracellular pathogens. On the other hand, Th2 cells produce IL-4, IL-5, IL-6, IL-9, IL-10, and IL-13 and induce an antibody response by downregulating B cells and activating eosinophils, thus inhibiting phagocytic cell activity [51].

## 5. OS and Inflammation during Pregnancy, Metabolic Changes in Chronic Diseases

Currently, gestational obesity and diabetes are associated with fetal disorders affecting the proteomic profile. An increase in markers of inflammation and OS is observed, as well as a decrease in the antioxidant system or impairment of vasorelaxation [68]. All of these characteristics could be linked to the appearance of neonatal hypoglycemia, respiratory distress syndrome, fetal macrosomia, platelet hyperaggregability, or the development of cardiovascular disease early in life [69,70]. Also, obesity is associated with a 1.3- to 3.8-fold increase in the incidence of preeclampsia or GDM [59], short-term hemorrhage, low APGAR score, congenital birth defects, and admission to neonatal intensive care [51]. Meanwhile, in the long term, an increased risk of cardiovascular disease and diabetes mellitus type 2 (DM2) in children [51,63,71,72].

### 5.1. Preeclampsia

Among the prominent hypertensive disorders in pregnancy is preeclampsia. It is a multifactorial disorder, one of the most common conditions during pregnancy on which hypertension (≥140/90 mmHg) and proteinuria (≥300 mg of protein in 24 h) are predominant, leading to dysfunction in several organs, poor placental development, and even maternal or fetal death [73,74]. Preeclampsia regularly develops from the placenta, and its early stages are known as placental syndrome. This syndrome is characterized by endothelial damage and dysfunction in different tissues accompanied by excessive free radical formation [75,76,77]. Hence, some potential OS markers such as ischemia-modified albumin (IMA), uric acid (UA), MDA, and metals like zinc (Zn), copper (Cu), and selenium (Se) have been used as diagnostic biomarkers for preeclampsia [10]. Inflammatory processes exacerbate the effects of preeclampsia, being IL-1α and IL-1β mainly secreted and expressed in the placenta. However, the involvement of other members of the interleukin family such as IL-18, IL-33, IL-37, and IL-38 has also been described [78]. Leakage of trophoblasts into the uterine wall accentuates inflammation and OS, leading to the presence of hypoxia and an increased placental ROS concentration [79]

Obesity, preeclampsia, and GDM pregnancy have an altered lipid metabolism, specifically related to low concentrations of lipoproteins and their lipid composition (free and esterified cholesterol, phospholipids, and triglycerides). Therefore, women with these conditions become susceptible to OS, and this results in an inhibition in the production of nitric oxide (NO). NO is an important vasodilator at the endothelial level, and it can lead to hypertensive disorders, ischemia, or stroke [80].

Increased inflammatory processes are observed from the first trimester in pregnant women at high risk of hypertension (Table 2), although, in the third trimester, a significant increase in proinflammatory cytokines and oxidative markers are observed in women who develop preeclampsia [81,82,83,84]. In late pregnancy, the inflammatory processes are characterized by elevated cytokines such as IL-1Ra, IL2, IL4, IL6, IL8, IL10, IL12p40, IL12p70, IL18, TNF-α [85,86,87,88], increased CRP [87,89,90,91], and increased adhesion molecules, such as the vascular cell adhesion molecule (VCAM-1), intracellular adhesion molecule (ICAM) [86,87,88], and proteins associated with endothelial dysfunction like the soluble L-selectin (sL-selectin) in the umbilical cord [87], and in obesity-associated inflammatory processes characterized by increased leptin [92,93].

OS during early pregnancy is characterized by increased thiobarbituric acid reactive substances (TBARS) and 8 isoprostane [82,83]. The increase in the lipid peroxidation marker, MDA, was observed from the second trimester, accompanied by a decrease in antioxidant defense measured by GSH levels as well as SOD and CAT activity [94]. However, during the third trimester and delivery, there is increased MDA [85,88,91,95], TBARS [87,96], xanthine oxidase activity, 8-isoprostane [89,92,97,98], and maintenance of CAT by SOD and GPx activities [95,99,100]. Enhanced antioxidant system activity reflects impaired redox state maintenance in the third trimester, and it is observed by increased lipid peroxidation and GSH/GSSG ratio.

**Table 2:** Markers of OS and inflammation in pregnant women with preeclampsia.

Study Groups	Oxidative and Inflammatory Changes	Effects in Mothers/Neonates	Ref.
*Follow-up from 1st to 3rd trimester*			
1st–2nd trim = 22 3rd trim = 11 >35 GW = 9	↑ HIF1A mRNA since 1st trimester in PW who later developed PE ↑ MIF, ENG, FLT1, and BACE2 mRNA at week 24–30, and only ENG remains high at 31–34 weeks in PW who developed PE	ND	[81]
Group I (risk of HAT without PE) = 82 Group II (risk of HAT developing PE) = 43	1st trimester in Group II vs. Group I: ↑ IL-6, TNF-α, IFNγ, TBARS, TAC 2nd trimester in Group II vs. Group I: ↑ hsCRP, TNF-α, IFNγ, TBARS, TAC 3rd trimester in Group II vs. Group I and 3rd vs. 2nd trimester: ↑ IL-6, hsCRP, TNF-α, IFNγ, TBARS, TAC	All parameters correlated with baroreflex sensitivity except BMI and TAC. TBARS had a significant independent contribution to BRS.	[82]
Follow up from 1st trimester to 3rd trimester n = 441	↑ CRP, TNF-α, and 8-isoprostane early in pregnancy (median 10 weeks) CRP of PE is similar to non-PE at the end of pregnancy ↑ TNF-α in PE across pregnancy and ↑ 8-isoprostane in PE in early gestation. No changes in IL-1, IL-6, IL-10 and 8-OHdG	50 PW developed PE The strongest associations were observed at approximately 18 weeks of pregnancy. TNF-α and 8-isoprostane were consistently elevated at all four time points in pregnancy. 8-OHdG was found as a protective factor against PE.	[83]
Follow up of 96 PW with high risk of PE at 16th week and next at 36th week	↑ MDA, hsCRP, and IL-6 at 36th week compared to 16th week of pregnancy ↓ Baroreflex sensitivity and NO	Decreased BRS and increased interleukin-6 are associated with reduction in NO	[84]
*Second trimester*			
PE = 49 Ctrl = 50	↑ MDA, Cardiac-specific troponin-I, and ↓ CAT, SOD, and GSH in PE vs. Ctrl	Association between SOD and MDA, and CAT with MDA. CAT correlated with hsCRP	[94]
*Third and delivery*			
PE = 20 Ctrl = 18	↑ 8-isoprostane in plasma only in post-partum ↓ urinary excretion of 8-iso-prostane in PE	The variation of urinary 8-iso-prostane depends on creatinine clearance and plasmatic endothelin-1	[97]
PE = 10 Ctrl = 11	↑ AOPP in PE vs. non-pregnant women but not with Ctrl ↑ CRP of PE vs. Ctrl	ND	[42]
PE = 19 Ctrl = 18 Non PW = 20	↑ Plasmatic and urinary 8-iso-prostane in PE and Ctrl vs. non-PW but not in PE vs. Ctrl ↓ α-tocopherol in PE and Ctrl vs. Non PW γ-tocopherol decreased in PE vs. Ctrl and vs. non-PW	ND	[98]
Mild PE = 47 Severe PE = 36 Ctrl = 50	↑ sFlt-1/PlGF, hs-CRP, 8-isoprostane and leptin and lower adiponectin in PE vs. Ctrl.	Significant positive correlations in sFlt-1/PlGF and hs-CRP or leptin. A weak inverse correlation emerged between sFlt-1/PlGF and adiponectin.	[92]
Non-pregnant controls = 10 PE = 28 Ctrl = 59	↑ Superoxide and ferritin in PE vs. control	During HP, superoxide concentrations correlate significantly with arterial stiffness, while in PE, superoxide is significantly correlated to microvascular endothelial function.	[101]
PE = 15 Ctrl = 28	↑ AGEs in PE vs. Ctrl	+ Correlation between circulating levels of AGEs and gamma-glutamyl transpeptidase, uric acid, glucose, insulin, and HOMA-IR in PE	[102]
Non-PW = 20 PW = 20 Ctrl = 20	↑ Serum resistin, vaspin, MDA, and IL-8 in PE vs. non-PE and Ctrl	Only resistin and vaspin were reduced in PE after four weeks of postpartum	[85]
Mild PE = 45 Severe PE = 40 Ctrl = 48	↓ TAC and ↑ TBARS, IL-6, TNF-α and IFN-γ in mild and severe PE groups compared to Ctrl	+ Correlation of circulating beta-human chorionic gonadotropin and OS and inflammatory markers	[86]
PE = 27 Ctrl = 43	MPO levels in placenta higher than the levels in normal control subjects	ND	[103]
PE = 5 Ctrl = 5	In preeclamptic women, intense xanthine oxidase immunoreactivity was present within the epidermis.	ND	[104]
PE = 67 Ctrl = 70	↑ CRP, fetal DNA, MDA and Hsp70 level	In PE serum, Hsp70 levels showed significant correlations with serum CRP, aspartate aminotransferase, and LDH activities MDA	[86]
PE = 53 Ctrl = 20 Mild PE = 32 Severe PE = 21	↑ hsCRP, IL-6, TNF-α and 8-isoprostane in PE vs. Ctrl and in severe PE vs. mild PE No changes in MDA	Plasma levels of 8-isoprostane were significantly correlated with the plasma levels of hs-CRP, IL-6, and TNF-α in patients with PE	[89]
PE = 71 Ctrl = 71	In amniotic fluid: ↑ sFLT1, sEndoglin, endothelin 1, and leptin in PE. sFLT1, sEndoglin, leptin, and adiponectin ↑ in PE IUGR than those without IUGR.	Leptin has the largest area under the curve (0.753). Amniotic proteins are involved in the inflammatory process of the human placenta.	[93]
PE = 44 Ctrl = 44	↑ serum CRP, plasma MDAin PE vs. Ctrl	Plasma OPN concentrations are increased in preeclamptic patients with extensive endothelial injury.	[95]
PE = 60 Ctrl = 60	↑ Hsp70, IL-1Ra, IL2, IL4, IL6, IL8, IL, IL10, IL12p40, IL12p70, IL18, INF&, IP10, MCP-1, ICAM-1, VCAM-1, sFlt-1, P1GF in PE vs. Ctrl	Elevated serum Hsp70 level and sFlt-1/PlGF ratio had a synergistic (joint) effect in the risk of preeclampsia	[86]
PE = 46 Ctrl = 42	↑ TBARS, TBARS/TAS, IL-6, TNF-a, a1-antitrypsin, CRP in plasma of PE vs. Ctrl ↑ CRP and a1-Antitrypsin in umbilical cord of PE vs. Ctrl ↑ sVCAM, sL-selectin in leucocytes of PE vs. Ctrl	+ Correlation between maternal and UCB TAS, IL-6, CRP in Ctrl and PE	[87]
PE = 33 Ctrl = 33	↑ IL6, MDA, CP, in plasma and placenta of PE vs. Ctrl ↑ TLR-4 and NF-kB in the placenta	ND	[88]
PE = 50 (17 mild and 33 severe) Ctrl = 33	↑ MDA in PE mild vs. PE severe ↑ IL-6, IL-10, TNF-a in PE vs. Ctrl ↑ IL-6/IL-10 ratio in PE vs. Ctrl	ND	[91]
PE = 18 Ctr = 18	↑ CAT, Il-6, TNF-α in PE vs. Ctrl Plasma Mg was positively correlated with CAT and GPx activities with concentrations of IL-6 and TNF-α	Plasma magnesium and urinary 8-isoprostane were associated with PE	[99]
PE = 100 Ctr = 50	↑ SOD, GSH/GSSG ratio, IL-6, and ↓ GSSG in placenta of PE vs. Ctrl	+ Associations between placental GSH levels with weight, head, and chest circumference and gestational age at birth	[100]

Abbreviations: 8-OHdG, 8-hydroxy-2′-deoxyguanosine; AOPP, advanced oxidation protein products; AGE, advanced glycation end products; BACE2, β-site APP-cleaving enzyme-2; BRS, baroreflex sensitivity; CAT, catalase; Ctrl, control group (pregnant women without preeclampsia); ENG, endoglin; FLT1, fms-related tyrosine kinase-1; GPx, glutathione peroxidase; GSH, glutathione; GSSG, oxidized GSH; GW, gestational week; HIF1a, Hypoxia-inducible factor 1-alpha; hsCRP, high sensitivity C Reactive Protein; Hsp70, heat shock proteins; ICAM-1, intracellular adhesion molecule; IFNγ, interferon gamma; IL, interleukin; IUGR, fetal intrauterine growth restriction; MCP1, monocyte chemotactic protein 1; MDA, malondialdehyde; MIF, macrophage migration inhibitory factor; MPO, myeloperoxidase; NF-kB, nuclear factor kappa B; NO, nitric oxide; OPN, osteoponin; PE, preeclampsia; PlGF, placental growth factor; PW, pregnant women; sFlt-1, soluble fms-like tyrosine kinase receptor-1; SOD, superoxide dismutase; TAC, total antioxidant capacity; TBARS, thiobarbituric acid reactive substances; TLR-4, toll-like receptor; TNF-α, tumor necrosis factor alpha; trim, trimester; VCAM-1, vascular cell adhesion molecule; VEGF, vascular endothelial growth factor.

### 5.2. Obesity

A common condition observed is the accumulation of lipids during the first trimester that decreases towards the end of the third trimester. Conversely, lipolysis decreases in the first trimester and increases by the third trimester. Similarly, glucose tolerance and insulin resistance behave in the same way, respectively [105]. Moreover, there is a high variability in lipid, protein, and carbohydrate metabolism, which are regulated by immunometabolic and immunoinflammatory processes, including proinflammatory cytokines and ROS production [106,107]. Nevertheless, when antioxidant–ROS balance is disturbed, extreme OS occurs, leading to abnormal placentation with increased vascular resistance inside the placenta, thereby allowing OS to overwhelm antioxidants, to shorten telomeres, and, ultimately, to lead to cellular senescence [108]. Also, when pregnant women develop preeclampsia, the processes of apoptosis, autophagy, and cellular senescence, which play a critical role in placental and fetal homeostasis and growth, appear disrupted, increasing susceptibility to negative conditions in the fetus and the mother [109]. Among the conditions that present an alteration in metabolic and inflammatory processes is obesity, which is a chronic disease considered a risk factor for the development of preeclampsia, GDM, anemia, complications or death in childbirth in women, increased cardiovascular risk, insulin resistance, hyperinsulinemia, and prevalence of inflammatory processes in the neonate [110,111,112]. Usually, pregnant women with obesity at pre-pregnancy or becoming obese have a lipotoxic placental environment leading to increased OS and elevated concentrations of TNF-α, IL-1, IL-1β, IL-3, IL-4 and IL-6, and IFN-γ. These cytokines also regulate the activity and expression of placental fatty acid transporters, and, during the second or third trimester, they downregulate some metabolic pathways then increase the levels of total cholesterol, triglycerides, and very low-density lipoproteins (VLDL) [112,113,114].

Clinical studies show increased lipid peroxidation (MDA, LOOH) [115,116,117,118,119] and significant changes in antioxidant status (total antioxidant capacity and antioxidant enzyme activity) in pregnant women with obesity [119,120,121,122,123] during the third trimester of pregnancy (Table 3). Another study has shown an increase in lipid peroxidation and protein carbonylation (PC) in pregnant women with obesity versus controls, only in the third trimester, but not in the first and second trimesters of pregnancy. However, an increase in TNF-α was observed at 12–13 weeks of gestation, also in women with obesity who developed GDM. The ratio of reduced oxidized glutathione (GSH/GSSG) is an indirect marker of redox status [115]. In addition, increased antioxidant activity of SOD and CAT was observed in the placenta [122] and umbilical cord [119] of obese women compared to non-obese women, and compared to plasma activities during the third trimester of pregnancy. These results reveal necessary changes to maintain the homeostatic redox state in the placenta and the umbilical cord at the expense of maternal plasma redox. Inflammatory conditions have also been associated with reduced adiponectin levels in the third trimester [117,118,119,120,121,122,123,124]. Maternal BMI has been shown to have a significant positive correlation with increased levels of CRP, PC, GSSG/GSH, IL-6 ratio, and salivary CT [117,120,121]. These associations could be affected by hyperglycemia [113,116,119] and maternal age [115]. The association of mother and newborn in inflammatory and oxidative statuses revealed a positive correlation in the cord SOD activity of newborns with an increased SOD activity of normoglycemic ones. MDA levels in newborns were lower in term neonates and those with mothers who consumed vitamin supplements [119].

### 5.3. Gestational Diabetes Mellitus

The most frequent endocrinological complication in pregnancy, worldwide, is obesity, and it plays an important role in the development of GDM. Also, it is related to negative neonatal and obstetric conditions in the mother and infant at both cardiovascular and neurological levels [125,126]. Commonly, GDM is defined as an abnormal glucose tolerance that results in the development of hyperglycemia during pregnancy, and it can be triggered by obesity, a variation in adipokine production, and other external conditions, including maternal age, a diet rich in saturated fatty acids, family history of diabetes, and hypertensive processes during pregnancy [127]. The molecular pathophysiology of GDM has been extensively investigated. Proteomic studies in plasma from women with GDM have revealed changes in inflammation and OS markers, insulin resistance, blood coagulation, and lipid homeostasis during the second trimester of pregnancy, as well as elevated CRP in the first trimester of women who developed GDM [128] (Table 4).

Inflammatory processes are a key factor in the development of GDM, as increased activity or expression of inflammatory markers negatively alters insulin receptors, resulting in insulin resistance [129]. In addition to TNF-α, IL-6, adiponectin, resistin, or leptin that are involved in obesity, other biomarkers are included, such as the retinol-binding protein-4 (RBP-4), visfatin, adipocyte fatty acid-binding protein (AFAABP), and some novel proteins like visceral adipose tissue-derived serpin A12 (vaspin) [130], apelin [131], and omentin, which may be involved in the development of GDM and in the progression of obesity [132].

During the second trimester of pregnancy, OS and inflammation are established in GDM by an increase in F2 isoprostanes [133,134], ROS in lymphocytes [135], total OS, and OS index in serum [134], as well as an increase in the acute phase reactants, which are independent determinants of GDM after adjustment for BMI [135,136] (Table 4). At the end of pregnancy, there is an increase in antioxidant enzymes, like CAT and GSR in placenta [137]. In contrast, other studies did not show an increase in protein carbonylation [138] or TBARS [139]. However, in studies with a bigger sample size, an increase in MDA, hsCRP, and IL-6 in the plasma of pregnant women with GDM was observed to be associated with stress and postnatal depression [140].

Both GDM and obesity complications constitute independent risk factors that increase problems during delivery and that are associated with an increase in monocyte count in cord blood and with the expression of several genes like the silent information regulator sirtuin 1 (SIRT1) and uncoupling protein 1 (UCP1) in obese pregnant women [139], while growth/differentiation factor 15 (GDF-15) is increased in GDM [141]. Also, an increase in mitochondrial DNA levels, but dysfunctional syncytiotrophoblast mitochondria with morphological abnormalities [142], has been observed in the placenta of obese pregnant women who developed GDM. These physical abnormalities could lead to impaired pregnancy outcomes and future hypertensive [143,144] and neurological disorders [145].

**Table 4:** Inflammation and OS markers in pregnant women with GDM.

Study Groups	Oxidative and Inflammatory Markers	Oxidative Stress Changes	Inflammation Changes	Effects in Mothers and Newborn	Ref.
GDM = 22 Ctrl = 22	Proteome profiling in plasma	↓ PON/LAC3 in GDM vs. Ctrl	↑ CRP and ↓ IGFBP2 in GDM vs. Ctrl	Strong correlations among the inflammation-related proteins and proteins of blood coagulation, lipid homeostasis membrane, and antioxidative enzymes	[128]
GDM = 11 Ctrl = 23	Six F2-isoprostanes isomers	↑ 8-iso-15(R)-PGF2α levels in GDM vs. Ctrl	ND	Delta-6-desaturase (D6D) activity index, calculated using fatty acid ratios, was 9% lower in pre-existing diabetes than in controls	[146]
GDM = 48 Ctrl = 46	Total oxidative stress (TOS), total antioxidant status (TAS), and oxidative stress index (OSI) in serum	↑ Serum OGTT, OSI, TOS in GDM vs. Ctrl	ND	ND	[134]
GDM = 60 Ctrl = 75	CRP, NLR and PLR, PCT, and VAP-1	ND	↑ CRP, NLR, PLR, PCT and VAP-1 in GDM vs. Ctrl	+ Correlation of VAP-1 with glucose, HbA1c, PLR, and CRP	[136]
GDM = 114 Ctrl = 114	ROS (DCFH-DA) and DNA damage (comet assay) in lymphocytesNLR and MVP in blood	↑ ROS and DNA damage in GDM vs. Ctrl	↑ NLR and mean platelet volume (MPV) in GDM vs. Ctrl	The elevated parameters are independent determinants of GDM after adjustment for BMI	[135]
GDM = 30	CRP, ferritin, TNF-α, methylglyoxal, glycated albumin	ND	↑ PAF and TNF-α after diet ↑ MGO in GDM vs. non-pregnant healthy people	+ Correlation of MGO levels with HbA1c, pregnancy weight and HOMA-IR at GDM diagnosis and after 12 weeks	[147]
GDM = 40 Ctrl = 40	GDF-15 in serum	ND	↑ Serum GDF-15 levels in patients with GDM vs. Ctrl	+ Correlation of GDF-15 and BMI for both GDM and Ctrl ↓ Apgar scores at 1 min and 5 min in the GDM vs. Ctrl	[141]
GDM = 176 Ctrl = 164	MDA, hsCRP, IL-6, and NO in plasma	↑ MDA and ↓ NO in GDM vs. Ctrl	↑ hsCRP and IL-6 in GDM vs. Ctrl	Significant association of HbA1C, MDA, and interleukin-6 with maternal stress and postnatal depression	[140]
GDM = 17 Ctrl = 23	CAT, GPX, GSR, and SOD gene expression in placenta and omental and subcutaneous adipose tissue	↑ CAT and GSR in placenta of GDM vs. Ctrl No changes in GPx or SOD in placenta and no changes in adipose tissue	Treatment with hypoxanthine/xanthine oxidase-stimulated cytokine release (IL1B, MIP1b, and TNF-α) and TNF-α mRNA expression	ND	[137]
GDM = 8 T2D = 3 T1D = 12 Ctrl = 13	IL-8, MCP-1, and CP in plasma and umbilical cord	No changes in CP in mothers with DM vs. Ctrl	No changes in MCP-1 and IL-8 in mothers with DM vs. Ctrl ↑ MCP-1 and CP in umbilical cords from mothers with DM	MCP-1 correlated with ketone bodies and acetoacetate	[138]
GDM = 69 PWOb = 44 PWOw = 48 Ctrl = 104	TBARS in placenta and UCP2, SIRT1, PPARα, TLR4, and GR-a gene expression in placenta tissue	↑ UCP2 expression in PWOw and PWOb vs. Ctrl	↑ SIRT1 expression in PWOw and PWOb vs. Ctrl ↑ GR-a expression in GDM vs. Ctrl	↑ Monocyte count was higher in the cord blood PWOb with normal glucose tolerance ↑ Serum leptin PWOb compared to Ctrl and their offspring	[139]

Additional abbreviations: GDF, growth differentiation factor-15; GSR, glutathione reductase; IGFBP2, insulin-like growth factor-binding protein 2; MGO, methylglyoxal; MVP, mean platelet volume; NLR, neutrophil to lymphocyte ratio; PAF, platelet-activating factor; PCT, procalcitonin; PLR, platelet to lymphocyte ratio; PON, paraoxonase; PPARα, peroxisome proliferator-activated receptors; SIRT1, silent mating type information regulation 2 homolog; UCP2, uncoupling protein 2; VAP-1, vascular adhesion protein-1.

### 5.4. Risk of Preterm Delivery

OS and inflammation induce impairment of placental permeability, producing a hypoxic placenta, which usually leads to activation of the maternal systemic inflammatory response [148]. As a consequence, it affects both maternal and placental functions, thereby causing intrauterine growth restriction (IUGR), preterm delivery, gestational diabetes, preeclampsia, and aortic dissection during pregnancy [61,149]. Likewise, there is dysregulation in lipid profiles such as sphingomyelin and phosphatidylcholine, which have been associated with inflammatory biomarkers such as TNF-α, IL-6, and CRP [150].

### 5.5. Non-Alcoholic Fatty Liver Disease (NAFLD)

NAFLD is regarded as a hepatic manifestation of metabolic syndrome (MS), which is characterized by elevated levels of saturated fatty acids, polyunsaturated fatty acids, and reduced levels of phospholipids in both the serum and HDL, specifically according to their PUFA fraction.

In both animals and humans, studies have established a relationship between adaption to maternal malnutrition (over or undernutrition), environmental factors, maternal stress, pathology genetics, and epigenetic modifications and early-life and later development of components of MS in offspring, including obesity, insulin resistance, impaired glucose tolerance, dyslipidemia, low HDL-C, increased VLDL-TG, and abnormal liver function such as NAFLD [151,152]. Moreover, recent studies on young adults revealed that being born to an obese mother induced perturbations in adipose tissue function and lipid homeostasis, increasing the risk for developing NAFLD in adulthood three-fold [153].

Declining adipose tissue function is a key characteristic in the transition to metabolically unhealthy, hypertrophic obesity. Dysfunctional adipose tissue is characterized by chronic inflammation that may promote injury, increasing oxidative stress, insulin resistance, dysregulated lipolysis, and eventually lipotoxicity combined with ectopic deposition in peripheral organs such as the liver. Therefore, adiposopathy is a key trigger for the onset of obesity-associated hepatic steatosis [154,155].

In this context, exposure to an abnormal intrauterine milieu may be an important risk factor for the development of cardiometabolic diseases in childhood and adulthood. This adaptation to the intrauterine environment has been explained with Barker’s theory, also known as the intrauterine programming effect, where the intrauterine environment alters the metabolism of the fetus, redistributes its blood flows to protect important organs such as the brain, and even adapts to slower growth to decrease its substrate demands. On the other hand, those changes can be permanent in the structure and function of the offspring. According to this theory, the growth and development of the fetus are determined by three factors: first, the mother’s nutritional status; second, placental function; and third, the ability of the fetus to utilize nutrients [156].

## 6. Lipids and Lipoproteins in Pregnancy

Lipid metabolism during pregnancy becomes relevant since lipid concentrations change according to maternal requirements and fetal growth; subsequently, dysregulation of lipid metabolism is associated with endothelial dysfunction or immunological changes [150], while major alterations are found in the concentration of triglycerides and cholesterol and in the number of LDL and HDL particles [157], hyperlipidemia being a common condition even in normal pregnancy that allows glucose and calories to be utilized by the fetus. Nevertheless, it has been reported that maternal lipid levels during pregnancy are significantly correlated with the lipid profile of children during the first years of life [158]. Additionally, it is well known that lipid dysregulation is an important risk factor associated with the development of preeclampsia and cardiovascular disease in pregnancy.

### 6.1. Lipoproteins

**Figure 2:**
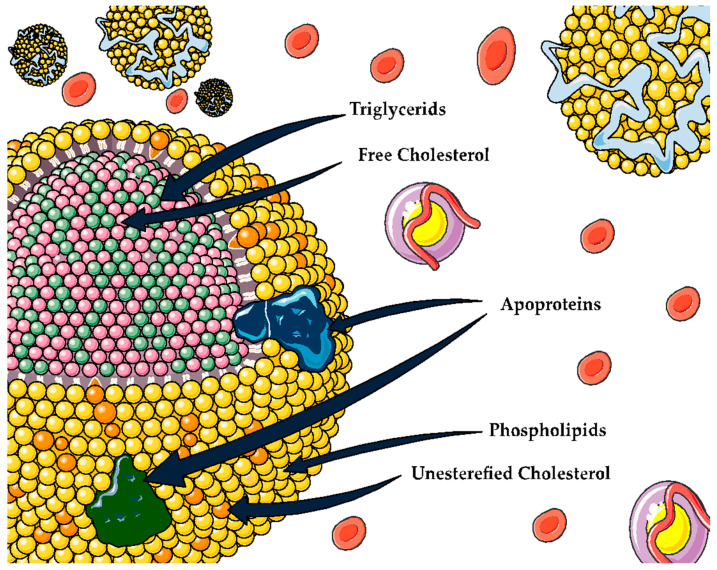
General structure for lipoproteins.

Lipoproteins are macromolecular complexes composed of hydrophobic lipids such as triglycerides and cholesterol esters on the inside, whereas their surface is formed by amphipathic lipids like phospholipids and free cholesterol. Moreover, there are proteins, known as the apolipoproteins (Apo), providing stability to the surface and conferring part of their own properties [5] (Figure 2). In recent decades, the role that certain lipoproteins play in different chronic-degenerative diseases has sparked interest, especially high-density lipoproteins (HDL).

### 6.2. HDL

High-density lipoproteins (HDL) are complex and heterogeneous structures constituting a lipid transport mechanism. Different components (lipids and proteins) of HDL are continuously being exchanged. As a result, they modify the composition, charge, and size of these particles. Currently, it has been described that HDL particles can similarly transport other compounds (about 250), such as sphingosine-1-phosphate, paraoxonase-1 (PON1), acute phase proteins (SAA) [159], platelet-activating factor acetylhydrolase (PFA-AH) enzymes and proteins, such as cholesterol ester transporter protein (CETP) and phospholipid transporter protein (PLTP), among many other components [160,161].

In this context, HDL has been attributed to exert some cardioprotective properties, including reverse cholesterol transport and antioxidant, anti-inflammatory, and antiatherogenic activities (Table 5) [162,163,164,165]. Many of these functions are important for a healthy pregnancy and good neonatal outcomes [166,167].

These positive effects are explained by the structure and chemical composition of these particles. However, it has been shown that these lipoproteins can lose or reduce their cardioprotective capacity, giving rise to prooxidant, proinflammatory, and proatherogenic lipoproteins, contributing to the process of atherosclerosis; this phenomena has been termed “dysfunctional HDL” [173,174].

Some study groups proposed the hypothesis that HDL delivers lipids to cells. For instance, Pérez-Mendez et al. demonstrated that HDL delivers cholesterol and sphingomyelin to endothelial cells in vitro [175]. Therefore, the possibility of the regulation of these lipoproteins on cell function after internalization and the delivery of their content is extremely high. Hence, the lipid delivery of HDL to cells becomes of particular importance when cell membranes should be intensively synthesized or re-structured, i.e., during fetal growth. HDL-C plasma levels and composition may change drastically during inflammatory processes.

**Figure 3:**
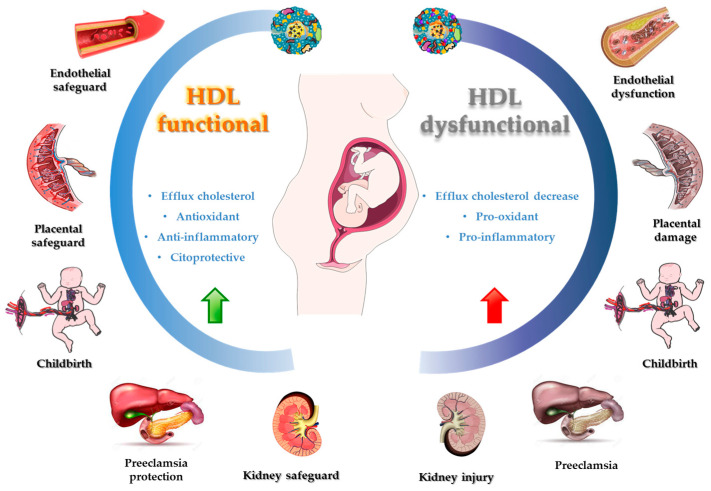
Functional and dysfunctional HDL during pregnancy.

It has been described that HDL can inhibit the oxidation of other molecules, such as LDL through free radical damage, which results in the generation of oxidized lipids with pro-inflammatory activity [176]. Nonetheless, in certain conditions such as obesity, diabetes, and other cardiovascular diseases, it has been observed that HDL loses its protective properties, becoming dysfunctional HDL [168,169,170], and leads to an increase in inflammatory processes and OS in several conditions, including pregnancy (Figure 3) [166,167].

Given that this is of great relevance in chronic degenerative diseases, the use of bioactive compounds from fruits, vegetables, foods of animal origin, and plants has become an alternative for improving the functionality and chemical composition of HDL. Thus, they are able to regulate the negative effects caused by OS and inflammatory processes.

### 6.3. HDL Role and Upregulation Contribution in Inflammatory Processes and OS

Pregnant women normally experience physiological changes, involving carbohydrate and lipid metabolism, insulin resistance, inflammation, coagulation, and OS, all of them causing endothelial damage [170]. Despite this unfavorable environment, pregnant women have better vascular function. Likewise, during embryogenesis and fetal development, the levels of apolipoproteins, lipoproteins, and lipids increase significantly. HDL-C levels change during pregnancy: in the first trimester, changes are insignificant, but, in the second trimester, these changes increase and then slightly decrease in the third trimester. In chronic inflammatory processes, the functional activities of HDL are reduced, the formation of new particles decreases, and catabolism increases. Also, structural changes occur at the protein level, such as the replacement of PON1 or Apo A1 molecules by proinflammatory proteins, including ceruloplasmin and SAA [177,178] that converts HDL to HDL-proinflammatory and results in increased chemoattractant activity, oxidation of LDL, and the release of additional proinflammatory molecules [179,180]. In this chronic inflammation, HDL-proinflammatory may accelerate immune responses toward pathogens, due to HDL remodeling. It is well known that immunological changes occurring in pregnancy for improved fetal tolerance lead to an increased susceptibility to infections. In the acute phase response, HDL levels decrease constantly, with an increase in SAA and ceruloplasmin concentration and a respective decrease in PON1 and Apo A1. Consequently, this could be one of the major risk factors during pregnancy (in trimesters of increased inflammatory processes) for the development of diseases or negative conditions.

Furthermore, an important role of HDL in pregnancy has been reported in the reducing of OS levels, both at placental level and in umbilical cord blood, which are mainly associated with PON1 activity [181]. An important factor in pregnancy is the higher activity of lipoprotein-associated phospholipase A2 (LpPla2) (mainly LDL and HDL), which is an enzyme synthesized predominantly by macrophages and associated with inflammatory processes and higher triglyceride levels, as well as in conditions of elevated OS such as GDM concentration of LpPla2, which is highly elevated compared to healthy women. However, this enzyme can be associated with HDL because it improves the antioxidant and anti-inflammatory functions of HDL, thereby reducing OS levels in plasma [182].

Another important complication of pregnancy caused by the increase in OS is preeclampsia, which affects both pregnant women and newborns and presents oxidative alterations in both LDL and HDL, caused by lipoperoxidation and inactivation of PON1, potentially leading to improper placentation [183].

It has been shown that a maternal diet rich in saturated and trans fatty acids causes harmful changes in the bacteria that colonize the intestine of the offspring, and these in turn produce metabolites that can subsequently affect different organs. Organic acids produced by intestinal bacteria may be involved in inflammatory mechanisms and play a key role in changes in the metabolism and develop neonatally or in adulthood. A high-fat diet during perinatal life predisposes greater expression of the NF-κB gene, which is a transcription factor of multiple biological processes, including immune and inflammatory responses and cell growth and survival [184,185].

Several studies have suggested that HDL dysfunction is a common pathological factor that connects the metabolic syndrome to NAFLD and cardiovascular disease development. The composition and structure of HDL particles seem to be characterized by the depletion of polyunsaturated fatty acid phospholipids and enrichment of saturated fatty acid ceramides [151,186]. In this context, preclinical studies have provided mechanistic insights as to how PUFA (especially essential fatty acids, EFA) deficiency promotes hepatic steatosis. EFA can negatively modulate the hepatic de novo lipogenesis machinery toward the negative modulation of the liver X receptor (LXR) of SREBP-1 and/or of the carbohydrate response element binding protein (ChREBP). Also, PUFA can activate the peroxisome proliferator, activated receptor-alpha (PPARα), and may promote fatty acid oxidation [186]. This is strong evidence for the role of PUFAs in modulating hepatic lipid metabolism [151].

### 6.4. Bioactive Compounds and HDL Functionality

Bioactive compounds have been widely studied as mediators of inflammation and OS in several conditions and diseases. Nevertheless, their mechanism of action remains unclear. Some studies describe that they are an important part of the secretion of inflammatory molecules (cytokines, adipokines, etc.), of the mediation of metabolic pathways, or of the regulation of gene expression at the muscle or adipose tissue level [187]. Currently, the most commonly studied bioactive compounds are folates [188], polyphenolic compounds [189], polyunsaturated fatty acids [190], prebiotics [191], and probiotics [192], along with their derivatives.

Studies have described that part of the functionality of HDL is linked to its chemical composition. This, in turn, depends on the appearance of some diseases causing HDL dysfunction [193], especially chronic degenerative diseases, which may also change HDL size as well as the number of circulating particles. In DM and coronary heart disease patients, it has been reported that, in addition to Apo A concentration modifications, the presence of OX increases glycoxidation and peroxidation of protein and lipid fractions of HDL, respectively [194,195,196,197].

An alternative way to reverse the mentioned effects is via bioactive compounds from functional foods. It is well documented that foods, for example, fruits and vegetables, fish, legumes, cereals, red wine, and elements of the Mediterranean diet, increase the concentration of HDL, TRC, and antioxidant activity at the same time, increasing the activity and/or expression of paraoxonase-1 (PON1), an atheroprotective enzyme that is bound to HDL [198,199,200]. Likewise, it has been reported that foods rich in polyphenols, hydrolysable tannins, and polyunsaturated fatty acids (PUFAs) modify the protein [201] c-HDL, Tg-HDL, and Phos-HDL content of HDL [197,198].

Alternative dietary modifications such as Mediterranean diet [202] seek to enhance HDL functionality via regulation of RCT. On the other hand, olive oil consumption [203] seems to have a similar effect but is attributable to its effect on the size and stability of HDL. For instance, a study based on red yeast rice extract and additional compounds shown to reduce cardiovascular risk in humans, a significant decrease in the lipid profile associated with cardiovascular risk, mainly c-LDL, and an increase in Apo A1 were found in 102 participants. However, there was no significant difference in the levels of c-HDL [204]. In contrast, a pilot study of 167 patients with metabolic syndrome features, bioactive compounds such as docosahexaenoic acid, β-glucans, and anthocyanins were proved as components of fortified functional foods, and a significant decrease in triglycerides and an increase in LDL-C were observed [205]. Moreover, in a study that evaluated the structure and function of HDL in adults with overweight, obesity, and cardiovascular risk, it was observed that there is a relationship between the decrease in inflammation markers such as IL-6 with sphingosine 1 phosphate (SP1) of HDL under a diet based on a Mediterranean diet [206].

Studies by our research group have demonstrated this: in an animal model as well as in women with acute coronary ischemic syndrome (ACS), by using a microencapsulated product enriched in antioxidants and PUFAs, mainly punicic acid, it was observed that this treatment improved the lipid profile, PON1 activity, and endothelial function mediated by HDL. In ACS women, the dysfunctionality of HDL was reverted by regulating the protein and lipid composition of the smallest subclasses (HDL3) [201,207]. Two possible explanations for these results suggest that bioactive compounds present in this microencapsulated product could remodel alterations in HDL under conditions of dyslipidemia, OS, and inflammation. Also, another possible explanation is that HDL acts as a vector for bioactive compounds, enhancing its bioavailability and potentially increasing its health benefits.

## 7. Conclusions

Health in pregnant women is of great relevance in preventing cardiometabolic diseases in offspring. In this context, the properties of HDL, like removing oxidized lipids, carrying bioactive compounds, inhibiting expression and activity of pro-inflammatory molecules, lipid transport or regulation of lipid metabolism, are important during pregnancy to progress to childbirth without complications, in addition to promoting placentation and healthy development of the fetus. Moreover, processes of lipoperoxidation, glycoxidation, and the release of pro-inflammatory molecules, which may possibly be reverted by the interaction and transport of bioactive compounds that are present in foods, characterize diseases such as preeclampsia, diabetes, and gestational obesity during pregnancy. However, more studies are needed in order to find therapies that regulate metabolic processes to prevent diseases and to ensure a healthy pregnancy.

## Figures and Tables

**Table 1:** Pregnancy phases and inflammation.

Trimester	Cytokines	Clinical Outcome in the Mother	Clinical Outcome in the Fetus/Infant	Ref.
First Trimester (Proinflammatory)	IL-6	Infection and chorioamnionitis increased prevalence	Fetal inflammatory response disturbances Reactive airway disease Ectopic lipid accumulation in liver	[61,62,63]
	IL-8	Preeclampsia development	Insulin resistance Bronchopulmonary dysplasia	[51,61,63,64]
	TNF-α	Increases obesity and GDM risk	Adipose tissue alterations	[61,65]
	MCP-1	Increased risk of miscarriage and GDM risk	Undetermined	[61]
	RANTES G-SCF	Increased risk of miscarriage	Undetermined	[61]
Second Trimester	IL-4	Decreases the risk of preterm birth, preeclampsia Increased risk of systemic lupus erythematosus (SLE)	Undetermined	[61]
	IL-10	Decreases the risk of preterm birth and preeclampsia Increases obesity and GDM risk	Decreased expression of the inflammatory cytokine gene in the uterus and placenta	[61,66]
	IL-13	Development of fetal inflammatory response syndrome	Undetermined	[61]
Third Trimester (Proinflammatory)	IL-6	Increased risk of miscarriage, obesity, GDM, and preeclampsia risk Development of reactive airway disease (RAD)	Development of reactive airway disease (RAD)	[51,61,62,63,65,66,67]
	IL-8	Increased prevalence of infection, preeclampsia, and chorioamnionitis Development of fetal inflammatory response syndrome	Undetermined	[61,66]
	MCP-1	Increased risk of miscarriage, autoimmune diseases, and GDM	Undetermined	[61]
	RANTES-G-SCF	Increased risk of miscarriage	Undetermined	[61]
	TNF-α	Preeclampsia	Wheezing and lower respiratory tract infections. Intrauterine growth restriction	[61,63,67]

**Table 3:** Markers of OS and inflammation in pregnant women with obesity.

Study Groups	Oxidative and Inflammatory Markers	OS Changes	Inflammation Changes	Effects in Mothers and Newborns	Ref.
PW Ctrl = 28 PWOw = 26 PWOb = 26	MDA, LPH in plasma CP in RBCs Plasmatic IL-6 and TNF-α	↑ MDA, LOOH and CP in PWOb and PWOw vs. Ctrl in the 3rd trimester vs. the 2nd trimester	↑ TNF-α in PWOb vs. PW at 12–13 months of gestation ↑ TNF-α and IL-6 in women who developed GDM	+ Correlation of LOOH, MDA and CP with gestational age in all groups and higher levels of these markers in PW who developed GDM vs. PWOb in 2nd and 3rd trimesters	[115]
Ctrl = 15 PWOb = 15	Serum iron and transferrin saturation (Tsat), CRP, IL-6, GSH, and GSSG	Ratio of serum GSSG/GSH was higher in the PWOb compared to Ctrl	↑ TNF-α in PWOb vs. Ctrl. No changes in IL-6 and iron	+ Correlation between maternal BMI and CRP. - Correlation between BMI and iron status in cord blood. Serum iron and Tsat were found to be significantly lower in cord blood from PWOb compared to Ctrl	[120]
Ctrl = 40 PWOb = 40	Serum MDA and hsCRP at 28 weeks of gestation	↑ MDA and glucose after 3 h OGTT were higher in PWOb No changes at baseline.	Higher levels of hsCRP at baseline and after 3 h of OGTT in PWOb vs. Ctrls	After the OGTT at 28 weeks of gestation, 16 patients in the obese group and 1 in the control group had GDM.	[116]
Weight gain Insufficient, IWG = 28 Normal, NWG = 20 Excessive, EWG = 26	Serum LOOH, MDA, CP, 8-oxodG, adiponectin, leptin, and resistin	No significant differences in LOOH, MDA, and CP concentrations between groups. A trend to lower 8-oxodG in PWOb	↓ Adiponectin in EWG + Correlation between Adiponectin and 8-oxodG concentration in NWG. In EWG, leptin was associated with LOOH, MDA, and CP.	ND	[124]
Ctrl = 25 PWOw = 21 PWOb = 22	Adiponectin, leptin, and resistin in serum. MDA, CP, and 8-oxodG in plasma	↑ MDA and CP in PWOb vs. Ctrl	↓ Adiponectin and resistin in PWOb	BMI estimating adiponectin and CP concentrations (37%). Gestational age predicts resistin and MDA (34%).	[117]
Ctrl = 15 PWOb = 15	CRP, GSSH, GSH, TNF-α and IL-6 levels in serum and cord	↓ GSSG/GSH ratio in PWOb vs. Ctrl	↑ CRP and IL-6 in PWOb vs. Ctrl. ↑ CRP in cord of PWOb vs. Ctrl	Maternal BMI correlated with CRP, GSSG/GSH ratio, and IL-6. Infants born from PWOb mothers did not have higher measures of OS.	[120]
Ctrl = 50 PWOb = 40	Nitric oxide (NO), CAT, SOD, GSH, CP and MDA in plasma and placenta	↓ SOD and CAT and GSSG levels in PWOb and their newborns vs. Ctrl ↑ MDA, CP, Nitrite and Red-NTB in plasma PWOb and their newborns vs. Ctrl ↑ CAT, SOD, GSH, MDA and CP in placenta of PWOb vs. Ctrl		+ Correlation between maternal and fetal levels of TG and plasma and placental MDA. Maternal nitrite and reduced NBT levels were significantly correlated with those of newborns of PWOb	[118]
Ctrl = 27 PWOb = 18 PW with obesity and GDM (ObGDM) = 17	TAC and CRP in saliva CRP in plasma	↑ TAC in saliva in ObGDM vs. PW. + Correlation between TAC and CRP in saliva or plasma	↑ CRP in saliva and plasma in ObGDM vs. PW. ↑ CRP in plasma in PWOb vs. PW	TAC and CRP correlates positively with BMI and glycemia. A significant interaction between maternal BMI and periodontitis for s-TAC levels.	[121]

Additional abbreviations. CP, carbonylated proteins; EWG, excessive weight gain; LOOH, lipohydroperoxides; PWOb, pregnant women with obesity; PWOw, pregnant women with overweight; red NBT, reduced nitroblue tetrazolium.

**Table 5:** HDL main functions.

HDL Function		Proteins and Lipids Associated with HDL Function	Ref.
Reverse cholesterol transport	HDL promotes cholesterol efflux from various cell types.	ABCAI, ABCG1, SR-BI, cubilin, ApoE receptor	[168,169]
	Removing excess cholesterol from lipid-laden macrophages is a crucial process in HDL-mediated vascular protection.		
Oxidant	HDL has antioxidant properties whereby it can remove and inactivate lipid peroxides from LDL and cells.	PON 1, Apo AI, PAF-AH, LCAT, Apo M, S1P, phospholipids	[159]
Inflammation	Controlling the activation of monocytes, preventing macrophage migration, and inhibiting the oxidation of LDL by blocking the 12-lipoxygenase that produces lipid hydroperoxides and leads to the oxidation of the LDL	VCAM, ICAM, TNF-α, SAA, ceramides	[169]
Vascular function	Modulation of endothelial nitric oxide synthase (eNOS) expression, leading to increased nitric oxide (NO) production and vasodilation	ABCA1, SR-BI, S1PR, S1P, Apo M	[170,171,172]

## Data Availability

Not applicable.
